# Understanding Pathogenesis and Care Challenges of Immune Reconstitution Inflammatory Syndrome in Fungal Infections

**DOI:** 10.3390/jof4040139

**Published:** 2018-12-17

**Authors:** Sarah Dellière, Romain Guery, Sophie Candon, Blandine Rammaert, Claire Aguilar, Fanny Lanternier, Lucienne Chatenoud, Olivier Lortholary

**Affiliations:** 1 Medical School, Paris-Descartes University, APHP, Necker-Enfants Malades Hospital, Infectious Disease Center Necker-Pasteur, IHU Imagine, 75015 Paris, France; sarah.delliere@gmail.com (S.D.); romain.guery@aphp.fr (R.G.); ag_claire@yahoo.fr (C.A.); fanny.lanternier@aphp.fr (F.L.); 2 Medical School, Paris-Descartes University, INSERM U1151-CNRS UMR 8253APHP, Necker-Enfants Malades Hospital, APHP, Clinical Immunology, 75015 Paris, France; sophie.candon@inserm.fr (S.C.); lucienne.chatenoud@inserm.fr (L.C.); 3 Medical School, Poitiers University, Poitiers, France; Poitiers University Hospital, Infectious Disease Unit, Poitiers, France; INSERM U1070, 86022 Poitiers, France; blandine.rammaert.paltrie@univ-poitiers.fr; 4 Pasteur Institute, Molecular Mycology Unit, National Reference Center for Invasive Fungal Disease and Antifungals, CNRS UMR 2000, 75015 Paris, France

**Keywords:** invasive fungal infections, mycoses, immune reconstitution inflammatory syndrome, fungal immunity

## Abstract

Immune deficiency of diverse etiology, including human immunodeficiency virus (HIV), antineoplastic agents, immunosuppressive agents used in solid organ recipients, immunomodulatory therapy, and other biologics, all promote invasive fungal infections. Subsequent voluntary or unintended immune recovery may induce an exaggerated inflammatory response defining immune reconstitution inflammatory syndrome (IRIS), which causes significant mortality and morbidity. Fungal-associated IRIS raises several diagnostic and management issues. Mostly studied with *Cryptococcus*, it has also been described with other major fungi implicated in human invasive fungal infections, such as *Pneumocystis*, *Aspergillus*, *Candida*, and *Histoplasma*. Furthermore, the understanding of IRIS pathogenesis remains in its infancy. This review summarizes current knowledge regarding the clinical characteristics of IRIS depending on fungal species and existing strategies to predict, prevent, and treat IRIS in this patient population, and tries to propose a common immunological background to fungal IRIS.

## 1. Introduction

The increasing prevalence of acquired immunodeficiency, subsequent to the human immunodeficiency virus (HIV) pandemic and medical advances, such as organ transplant, stem cell transplant, intensive anti-neoplastic chemotherapy, or immunomodulatory biological agents, has tremendously raised the prevalence of opportunistic infectious diseases, including fungal ones [1]. Further progress, such as anti-retroviral therapy (ART) in HIV patients, has managed to restore immunity, therefore shedding light on a new syndrome: immune reconstitution inflammatory syndrome (IRIS). IRIS is now known to occur during the course of various invasive fungal diseases (IFD). It can be defined as a clinical worsening, or the new presentation, of infectious disease after reversal of immune deficiency. This reversal can be driven by the introduction of ART in HIV patients, neutrophil recovery after chemotherapy and/or stem cell transplant, inadequate balancing of immunosuppressive therapy after solid organ transplantation (SOT) [2,3], and even by post-partum immunological changes after pregnancy [4]. IRIS is triggered by the recovery of immune cells, resulting in a “cytokine storm” and an exaggerated host inflammatory response. IRIS has been best described in HIV-infected patients as a syndrome occurring in the first 6 months of ART and associated with a wide range of opportunistic pathogens, such as JC virus, cytomegalovirus, non-tuberculous mycobacteria, *Mycobacterium tuberculosis*, cryptococci, and *Histoplasma* species [5]. IRIS is commonly divided into two clinical pictures. “Paradoxical” IRIS refers to a primarily diagnosed and treated infectious disease with a secondary inflammatory increase occurring during antimicrobial treatment and immunodeficiency reversal [6]. “Unmasking” IRIS refers to disease symptoms that first appear after immune recovery [6]. When occurring during fungal infections, these entities have been extensively studied in the context of cryptococcosis. Cryptococcal IRIS develops in 8-49% of patients with known cryptococcal disease before ART [6]. The panel and clinical presentation of IFD responsible for IRIS after immune recovery varies with the underlying primary or acquired immunodeficiency. It depends on multifactorial conditions, including the nature of the immune defect, host genetics, and fungal pathogenesis and exposure. Our understanding of IRIS’s pathogenesis remains poor. It is a true diagnosis challenge to distinguish IRIS from sole fungal infection or treatment failure due to a similar clinical presentation. Misdiagnosis and the resulting ineffective treatment with antifungals instead of anti-inflammatory drugs may result in the disease having a fatal course [7]. Furthermore, the therapeutics attempted for IRIS include many anti-inflammatory agents and biologic immunomodulators; however, these remain poorly codified in guidelines. In this review, we will summarize current knowledge on the risk factors and the clinical and biological manifestations of IRIS associated with various fungal infections. Current knowledge and cues to understanding the immunopathogenesis of IRIS will be detailed. Finally, IRIS management, including therapeutics and prevention strategies, will be discussed and research priorities highlighted.

## 2. Fungal-Pathogen-Associated IRIS Characteristics

### 2.1. Cryptococcus

The yeast *Cryptococcus* is the most frequently described genus in IRIS [6]. The difference between paradoxical and unmasking IRIS is also best described and defined for this pathogen [6]. In cases where cryptococcal disease was not recognized at ART initiation, it may be difficult to differentiate between IRIS (caused by the restoration of immune functions then called unmasking IRIS) and the progression of a disease in the context of persisting immunodeficiency. Therefore, Haddow et al. proposed a panel of criteria to support this controversial entity [6]. These criteria mainly include unusual, exaggerated, and heightened inflammatory features. The epidemiology of cryptococcal IRIS may describe various incidence rates. In a review, including 12 studies and 598 patients with diagnosed cryptococcal disease before ART initiation, paradoxical IRIS developed in 8–49% of patients [6]. Interestingly, it appears that the incidence is lower in high-income countries (i.e., 8% in France, 13% in Thailand) while the highest incidence is found in low-income countries (i.e., 49% in Uganda). Furthermore, it seems that cryptococcal IRIS is seen less frequently in the most recent studies [6,8]. The increase in access to improved antifungal therapies may explain such figures. The incidence of unmasking IRIS is much lower, ranging from 0 to 7%; however, a case definition has not been uniformly addressed. Cryptococcal paradoxical IRIS has been described in HIV, SOT (mostly in kidney and liver transplant recipients) [3,9,10], and in early post-partum after pregnancy [4]. IRIS may occur a few days or several months after ART initiation in HIV-infected patients. In SOT recipients, a mean occurrence of 6 weeks after the introduction of antifungal treatment has been described and is triggered by a reduction in immunosuppressive treatment [11]. In those cases, patients may experience allograft loss temporally related to the onset of IRIS through Th1 upregulation. IRIS seems to occur more frequently in patients receiving a combination of tacrolimus, mycophenolate mofetil, and prednisone than in patients receiving another immunosuppressive regimen [2,12]. Clinical manifestations are described in Table 1. IRIS non-specific symptoms may occur in organs that were not apparently initially infected by the fungus, resulting from exuberant tissue inflammation [13]. Diagnosis remains clinical and requires exclusion of other diagnoses, including worsening or relapse of infection, other opportunistic infections, tumors, and drug-related adverse effects. Cerebrospinal fluid (CSF) culture is typically sterile in IRIS; however, if IRIS arises shortly after antifungal treatment, culture may remain positive. In this case, comparing the fungal burden to CSF at the initial diagnosis of cryptococcosis can help to differentiate IRIS from relapse; however, this technique is not part of routine management. Moreover, it has been suggested that monitoring (1-3)-β-d-glucan (BDG) in CSF could be helpful [14]. Indeed, it has been shown in a recent Ugandan and South African cohort of HIV-infected patients with cryptococcal meningitis that BDG measured in CSF could contribute to the differentiation of fungal progression (i.e., positive BDG) from cryptococcal paradoxical IRIS (i.e., negative BDG) [14]. Furthermore, a PCR-based assay might also be useful: the FilmArray system was evaluated in 39 HIV-infected patients from Uganda with suspected cryptococcal meningitis and was able to detect *Cryptococcus* with 100% sensitivity and to distinguish relapse from IRIS in a limited number of patients [15]. IRIS-like syndrome has been described in immunocompetent patients. Cases have been described with both *Cryptococcus neoformans* and *Cryptococcus gattii* [16,17,18]. They report an extended overwhelming inflammatory immune response despite CSF culture sterilization with a poor prognosis and subsequent neurological sequelae. In one case, thalidomide was successfully used to decrease inflammation when corticosteroids were inefficient [17]. It is possible that antifungal therapy reduces the burden of *Cryptococcus*, thereby facilitating the reversion of a Th2 response to a Th1 response. However, all of these cases failed to study if the strains may belong to a hypervirulent clade and if a host immune polymorphism could have led to this specific clinical presentation.

### 2.2. Candida

Chronic disseminated candidiasis (CDC), also called hepato-splenic candidiasis, has been suspected to be a form of candidiasis-related IRIS [19]. This clinical entity develops in patients who recently experienced profound and prolonged neutropenia, especially at neutrophil recovery after chemotherapy for acute leukemia. Before the introduction of posaconazole as the primary antifungal prophylaxis, its incidence ranged from 3 to 29% in this population [19], and a diagnosis is usually obtained within 2 weeks following immune recovery but can sometimes be diagnosed as late as 165 days thereafter [19]. Symptoms, which are described in Table 1, usually persist despite antifungal therapy. MRI has a much better sensitivity than ultrasonography and computed tomography (CT) to detect micro-abscesses, which are most frequently localized in the liver and the spleen and result from an exuberant inflammatory response [20]. Positron emission tomography (PET) has been increasingly used and shows promising results in CDC diagnosis [21,22]. At a histopathological level, epithelioid granulomas and micro-abscesses are encountered in most cases [19]. Blood cultures are negative in more than 80% of cases, and microscopy shows the presence of the yeast in less than 50% of cases [19,23]. Biomarkers could be useful in diagnosing CDC. BDG are usually highly increased (>500 ng/L); however, serum detection of mannan antigens and antibodies (Platelia® Candida) appears to be more sensitive to detect the disease and monitor treatment efficacy [24]. Ongoing pathophysiological and imaging studies from our group aim to better decipher the CDC entity. To our knowledge, no form of paradoxical candida-related IRIS after ART initiation in HIV-infected patient has ever been reported.

### 2.3. Aspergillus

*Aspergillus*-related IRIS also occurs during neutrophil recovery, especially after a course of chemotherapy to treat acute leukemia, or after stem cell recipients [25]. The mean time to clinical and radiological findings of IRIS from an absolute neutrophil count >100/µL and >500/µL was 3.5 days and 2 days, respectively, in a cohort of 19 patients [26]. A few cases of *Aspergillus*-related IRIS have also been described in lung transplant recipients [27]. The clinical manifestations, which are summarized in Table 1, include non-specific worsening, or new onset, of hypoxia, chest pain, cough, dyspnea, and hemoptysis [26]. A CT scan can show a size increase in pulmonary infiltrates, a pleural effusion, nodular lesions, and/or cavitation of a pre-existing lesion [26]. As with all fungi-related IRIS, it remains a diagnosis of exclusion. However, a decrease in serum galactomannan together with a perceived clinical and radiological worsening is good supporting evidence [28]. Overall, *Aspergillus*-related IRIS in neutropenic patients appears to have a good prognosis [28].

### 2.4. Histoplasma

The dimorphic yeast *Histoplasma capsulatum* is represented by two species found in endemic regions: *Histoplasma capsulatum* variety *capsulatum* is mostly encountered in North and South America as well as a few regions in Africa and Asia, while *capsulatum* var. *duboisii* is found in Africa only. Immunocompetent patients infected with *Histoplasma capsulatum* are mostly asymptomatic (>90%), while in immunocompromised patients, a reactivation of infection is common and may lead to a disseminated disease associated with a poor prognosis. *Histoplasma*-related IRIS may present as “unmasking” or “paradoxical”. Most cases have been described with *H. capsulatum* var. *capsulatum* in HIV patients [29,30,31], solid organ transplant recipients [32], and patients receiving TNFα inhibitors [33]. To our knowledge, only one case was described with *H. capsulatum* var. *duboisii* in a HIV patient [34]. The HIV patient population has been the most studied. The incidence of histoplasmosis appears to be higher in patients treated with anti-TNF monoclonal antibodies (e.g., infliximab) than in patients receiving soluble TNF-α receptors (e.g., etanercept) [33]. Overall, IRIS occurs in 9.2% of this patient population [33]. The median time of onset of IRIS symptoms from TNF-α inhibitors discontinuation was 6 weeks (1–45) in 9 patients [33]. Clinical presentations are described in Table 1.

### 2.5. Pneumocystis jiroveci

*Pneumocystis jiroveci* is a unique fungal organism and was only recently reclassified from a protozoan to an ascomycetous fungus after an analysis of the ribosomal DNA (rDNA) subunit [35]. It is a common opportunistic fungal pathogen and causes pneumonitis in immunocompromised patients. While *P. jirovecii* transmission occurs via person-to-person contact during the first years of life and is controlled by the immune system, it may rapidly multiply in the lungs of immunocompromised patients and lead to severe hypoxia and death [36]. Cases of *Pneumocystis*-associated IRIS have been described mostly in HIV-infected patients and patients receiving high-dose corticosteroids secondarily tapered [37,38,39]. In an analysis of 15 reports, time to IRIS symptom onset following ART initiation was 15 days (3–301 days) [40]. *P. jiroveci* pneumonia (PJP)-IRIS presents as a recurrence of fever, dyspnea, cough, and night sweat in patients treated for PJP. When performed, a CT scan often shows a recurrence of ground glass opacities; however, many cases also report atypical radiologic manifestations, including nodules, consolidations, and organizing pneumonia [37,41,42]. No adequate diagnostic criteria have been described for this entity. Similarly to other fungi-associated IRIS, microbiological tests (a direct examination of the broncho-alveolar lavage, PCR) are more likely to be negative. Nowadays, a PJP diagnosis often relies on quantitative PCR performed on the bronchoalveolar lavage or induced-sputum [43]. However, the lack of consensus on molecular threshold values for fungal load makes the PCR results difficult to interpret, even more so in the case of IRIS. Furthermore, the lack of a culture technique for *P. jiroveci* creates an additional challenge to distinguish relapse from IRIS. When performed, especially in organizing pneumonia forms, biopsies also showed granulomatous inflammation [37,41,42,44]. These histopathological results have also been described in liver and renal transplant recipients; however, whether a decrease in immunosuppression was responsible or not for IRIS was unclear [45,46].

### 2.6. Other Fungi

Fungi-related IRIS has also been described during infections with *Talaromyces marneffei* (ex-*Penicillium marneffei*) [47,48,49,50,51], *Coccidioides* spp. [52], *Paracoccidioides* spp. [53], *Sporothrix schenckii* [54], *Fusarium* spp. [55], or the newly described *Emergomyces africanus* [56]. To the best of our knowledge, no case of IRIS has been described with any of the Mucorales and phaeohyphomycetes. All IRIS cases related to these more unusual fungi occurred in HIV-infected patients after introduction of ART except for the case involving *Fusarium*, which occurred after neutrophil recovery in a patient treated for acute myeloid leukemia.

## 3. Is There a Common Background for Fungal IRIS?

Finding a common pathophysiological explanation for all fungi-associated IRIS may appear to be impossible in the light of the diversity of organisms, clinical presentation, and immunological mechanisms underlying a sudden increase in immune response. Although all data come from an isolated clinical picture and a limited series, the histopathological hallmarks when a biopsy is performed seem to consistently involve numerous non-growing fungi, necrosis, macrophages, and, more specifically, granuloma (Table 1). However, objective histopathological data are lacking concerning *Aspergillus*-associated IRIS [26,28]. This granulomatous reaction is described as clusters of epithelioid macrophages, which are sometimes vacuolated and always CD68-positive [57,58]. A lymphocytic infiltrate can also be observed, predominantly at the periphery of granulomatous lesions. Hence, a common immunological explanation might link the underlying mechanisms of fungal IRIS in these very different clinical pictures.

**Figure 1:**
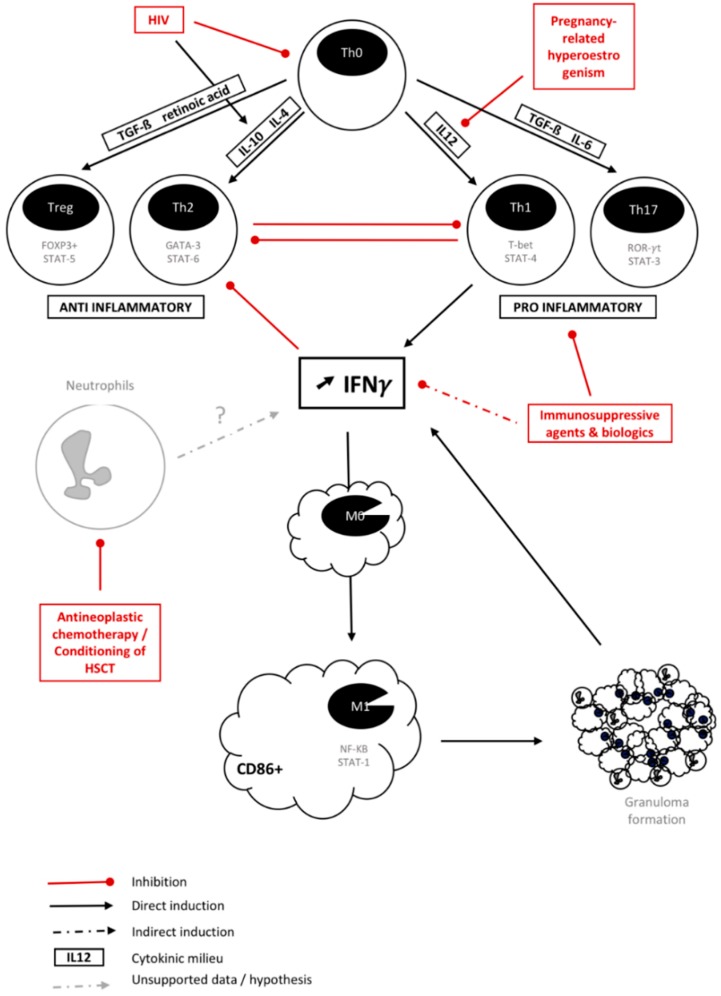
Proposed immune pathophysiology of IRIS. Precursor T helper cells (Th0) differentiate into Th1, Th17, or Th2 cells or Tregs according to cytokines produced in the surrounding milieu through induction of specific transcription factor expression: FOXP3/STAT-5, GATA-3/STAT-6, T-bet/STAT-4, and ROR-γt/STAT-3 for Treg, Th2, Th1, and Th17, respectively [60,61]. Th1 and Th17 cells are pro-inflammatory cells and produce IFN-γ driving macrophage differentiation into M1 macrophages and their activation. M1 macrophages promote granuloma formation and subsequently produce more IFNγ, thus creating an amplification loop leading to an inflammation burst [62]. HIV drives depletion of all Th cell subsets and favors an anti-inflammatory response in remaining cells through a change in cytokine balance [63,64]. Pregnancy hormones inhibit differentiation of naive Th0 cells into Th1 cells, thus promoting a Th2 environment [65]. Biologic agents, such as infliximab, inhibit the pro-inflammatory cytokine TNFα [66]. Immunosuppressive therapeutics inhibit Th1/Th17 response and allow graft acceptance in solid organ transplant [3]. Neutropenia resulting from chemotherapy or conditioning for hematopoeitic stem cell therapy leads to a decrease in IFNγ production through cytokinic interaction with T-cell production; however, data are lacking [67]. Many feedback loops exist between these different players depending on cytokine production. Sudden dysregulation at any level may create an exaggerated inflammatory response with an IFN-γ-unregulated increase leading to IRIS. FOXP3, fox head box P3; IFN, interferon; IL, interleukin; PMN, polymorphonuclear; TGF, transforming growth factor; ROR, retinoid orphan receptor; STAT, signal transducer and activation of transcription; HSCT, Hematopoietic Stem Cell Therapy.

An inadequate balance between pro-inflammatory Th1 response and anti-inflammatory Th2 response was commonly admitted to be the origin of IRIS. With the recent discovery of Th17 and regulatory T cell (Treg) responses, this model evolved to an inbalance between pro-inflammatory Th1/Th17 and anti-inflammatory Th2/Treg axes [3,59,60,61]. Cytokines driving the differentiation of naïve Th0 cells into Th1, Th2, Th17, and Treg stimulated by cytokines are summarized in Figure 1. Specific transcription factor pathways are detailed for each subset (Figure 1) [3]. IRIS is now believed to arise from an unregulated Th1/Th17 leading to increased production of interferon-γ (IFNγ) [3].

Overall, this exaggerated inflammatory response translates in radiological findings as edema and abnormal contrast medium uptake into pulmonary and brain lesions [58]. Cells of the innate immune system, such as monocytes, macrophages, and neutrophils, are of increasing interest in IRIS pathophysiology, since granuloma appears to be frequently found in IRIS lesions [19,41,47]. Indeed, granuloma is the histopathology hallmark in chronic disseminated candidiasis [19] and is commonly found in other fungi-related IRIS [37,41,48,49,56]. An extended description of granuloma in fungi-related IRIS is unavailable, and should be better studied in particular through immune staining to understand which cell types are implicated and their degree of differentiation/activation. Granulomas may display very distinct features, may be activated or latent, and their cell-type composition may vary according to the situation and pathogen [68]. In a simplistic approach, excess IFNγ produced by Th1 cells, neutrophils, or activated macrophages will elicit the differentiation of monocytes towards macrophages and activate their phagocytic activity as well as stimulate granuloma formation. IFNγ favors the classical activation of macrophages into M1-phenotype macrophages [62]. These macrophages secrete large amounts of pro-inflammatory cytokines, such as IL-1β, TNFα, IL-12, IL-18, and IL-23, driving in return a Th1/Th17 cell inflammatory response. Phenotypically, M1 macrophages express high levels of histocompatibility complex class II and the CD86 marker. M1 macrophages are implicated in initiating and sustaining inflammation, and can, therefore, be detrimental. Furthermore, M1 polarization appears to be predominant in granuloma formation [69]. Conversely, M2-polarized or alternately activated macrophages produce IL-4, IL-13, and IL-10 and are prone to immune regulation and tissue remodeling [70]. A further analysis would provide significant data to help us understand fungi-associated IRIS.

The immune pathophysiology appears to vary according to the underlying type of immunosuppression before immune recovery. HIV, in addition to depleting the total pool of CD4+ T-cells by apoptosis, including Th0 cells, induces preferential death of Th1 cells and differentiation of the remaining Th0 into Th2 cells through a critical change in cytokine balance [63,64]. Introduction of ART leads to an initial redistribution of CD4+ T-cells to the blood rather than proliferation. After 4–6 weeks, the production of naive CD4+ T-cells and memory T-cells coincides with the mean onset of paradoxical IRIS [71]. A concomitant infection responsible for increased circulating pro-inflammatory cytokines may also contribute to dominant Th1 differentiation and IRIS. In addition, HIV induces Tregs to migrate and accumulate in peripheral and mucosal lymphoid tissues [72]. Montes et al. have described an initial redistribution of Tregs to the blood at ART initiation [73]. This tissular depletion in Tregs may also participate in a locally uncontrolled inflammatory response. Moreover, the functional capacities of Tregs appear to be reduced [74].

After solid organ transplantation, graft survival relies on the inhibition of alloreactive Th1/Th17 responses by immunosuppressive drugs (i.e., mycophenolate mofetil, calcineurin inhibitors, corticosteroids…) through different mechanisms [3]. Calcineurin inhibitors, especially tacrolimus, strongly suppress the Th1 response while rapamycin promotes Treg survival and function and suppresses the differentiation of Th17 cells [3]. Corticosteroids decrease Th1 responses but also expand Th2 cells and Tregs [3]. Post-transplant IRIS is subsequent to a decrease in immunosuppression due to drug–drug interactions or an intentional modification in drug dosage in the context of an ongoing infection, therefore increasing Th1/Th17 responses. However, a recent study showed that only a discontinuation of calcineurin inhibitors influenced the development of IRIS in cryptococcosis by a 5-fold higher risk [11]. The excessive Th1 response observed in IRIS may also lead to collateral damage to the graft. Indeed, in a prospective study involving 54 renal allograft recipients with cryptococcosis, 5.5% of patients presented with IRIS and the renal graft was lost due to chronic rejection in 66% of patients with IRIS as compared to 5.9% of IRIS-free patients [12].

Immunomodulatory biologic agents are increasingly being used to treat chronic autoimmune and inflammatory conditions, such as rheumatoid arthritis and inflammatory colitis. Tumor necrosis factor (TNF)-α, a cytokine mainly produced by activated macrophages, is involved in systemic inflammation and plays a key role in the recruitment of immune cells and granuloma formation. Histoplasmosis is the most prevalent IFD in patients undergoing TNFα inhibitors therapy, followed by candidiasis and aspergillosis [66]. Most mycoses described in those patients were associated with the use of the monoclonal antibodies infliximab/adalimumab rather than the soluble TNFα receptor etanercept, reflecting the difference in the mechanism of action between those drugs [66]. Discontinuation of TNFα inhibitors results in IRIS in 9.2% of patients with histoplasmosis [33]. Alemtuzumab, a humanized CD52 monoclonal antibody that depletes T and B cell populations, has also been involved in fungi-related IRIS with *C. neoformans* and *P. jiroveci* upon its withdrawal [75,76].

A prompt recovery of neutrophils after a stem-cell transplant or a chemotherapy cycle has been linked to a severe pulmonary complication subsequent to inflammation during invasive pulmonary aspergillosis [25]. Likewise, chronic disseminated candidiasis manifests during neutrophil recovery [19]. Despite the lack of studies and data concerning this IRIS-like syndrome following the fast expansion of neutrophils after a stem-cell transplantation, it could be expected that a direct or indirect increase in IFNγ plays a role in pathogenesis. Indeed, a subset of neutrophils, Gr-1+/CD11b+ cells, has been shown in mice to produce IFNγ and mediate early graft loss or take part in severe renal ischemia reperfusion injury [67,77]. However, production of IFNγ by human neutrophils is controversial [78]. They may, nonetheless, participate through indirect mechanisms, especially through a crosstalk with Th17 cells, in cytokine production to increase IFNγ levels [79]. More data are needed to understand IRIS following neutrophil recovery, and its pathogenesis is currently being studied in the CANPHARI study (NCT01916057) headed by our group.

During pregnancy, mechanisms of fetal tolerance lead to downregulation of the Th1/Th17 axis. Interestingly, regulation of M2 macrophage polarization is required for successful pregnancy, and is sustained by pregnancy hormones, such as estrogen and HCG [65]. A shift in cytokine pattern is observed during the post-partum period that may be associated with pathological inflammatory syndrome and has been documented 3–6 weeks after delivery [4,80].

In addition to this immune dysregulation, commonly used antifungals are known to bear some immunomodulatory properties that could contribute to IRIS. These data only supported by in vitro studies, and the few in vivo studies in mice are to be taken with caution. Amphotericin B deoxycholate upregulates Th1 response through toll-like receptor 2 (TLR-2)-mediated transcription and inflammatory cytokine transcription [81,82]. Conversely, the lipid formulation of polyene has no effect on, or even downregulates, the inflammatory response directly due to the intrinsic properties of the liposomes [82]. However, it may confer an increase in the phagocytic properties of macrophages and neutrophils against numerous fungi [82,83,84,85]. Echinocandins unmask β-glucan from the fungal cell wall, eliciting pro-inflammatory cytokine release upon its recognition through pattern recognition receptors (PRR). Azoles seem to be the least active with respect to modulation of the host’s immune system [82]. However, fluconazole and voriconazole have been shown to enhance phagocytic pro-inflammatory activity through a TLR2 interaction [82]. Overall, the initial choice of antifungal could influence the subsequent inflammatory response; however, their immunomodulatory effects have not been studied in the context of IRIS.

Lastly, three more elements, often omitted and far less studied, may be discussed to explain IRIS’s pathophysiology: fungal strain immunomodulatory characteristics, host immune system features, and autoimmunity disorders. Firstly, *Cryptococcus* itself inhibits the Th1 response while inducing a Th2 response that compromises the host’s resistance in mice [86]. Furthermore, the cryptococcal genotype has been shown to influence the immune response and human clinical outcome after meningitis [87]. In addition, Desnos-Ollivier et al. showed that mixed strain infections are seen in 20% of patients and could drive overstimulation of the immune system [88]. Similarly, infection by *P. jiroveci* often results from mixed strain genotypes [89]. However, the impact of strain genotype in IRIS has not been studied. Secondly, on the host side, human immune system genetics is a growing field of interest and may be involved in IRIS development. Polymorphisms in cytokine genes may play a role in host susceptibility to IRIS [90]. For instance, a non-synonymous polymorphism in IL-23R is associated with a reduced risk of schistosomiasis-associated IRIS in a Kenyan population [91]. Similarly, a common haplotype of the IL-4 promoter was over-represented in patients with CDC [92]. Also, a single nucleotide polymorphism in the promoter of leukotriene A4 hydroxylase (LTA4H) regulating the balance between anti-inflammatory lipoxins and the pro-inflammatory LTB4 is responsible for a higher incidence of severe tuberculosis-associated IRIS [93]. One could easily hypothesize that polymorphisms of the human IFNγ receptor may lead to different susceptibility patterns to IRIS. Third, opportunistic infection results in tissue damage and the epitope spreading that is known to predispose to autoimmunity [71]. Furthermore, various autoimmune disorders have been discovered in HIV-infected patients after ART initiation that may play a role in the excessive inflammatory response [71]. Nevertheless, these hypotheses require further investigations, which are currently ongoing through a collaborative work about chronic disseminated candidiasis between the group of Lausanne and ours.

Overall, any rapid immune recovery can lead to IRIS, driven by multifactorial factors, such as patient response to drugs (ART, antifungals, …), host immune genetics, and the microbial strain. Fungal pathogen immunogenicity may trigger an overshoot of IFNγ production either through an enhancement of the Th1/Th17 axis and/or rapid neutrophil reconstitution leading to overproduction/activation of CD68+ macrophages and promoting granuloma formation and inflammatory disease.

## 4. A Limited Therapeutic Arsenal Against IRIS

The ideal management of fungi-associated IRIS in general remains unknown. No recognized clinical guidelines have been dedicated to this challenging subject matter except those from IDSA for the management of IRIS in cryptococcal disease [7]. They do not recommend a specific treatment for minor IRIS manifestations that usually resolve within a few days or weeks. However, for complications involving central nervous system (CNS) inflammation associated with increased intracranial pressure, they advise corticosteroids (0.5–1 mg/kg/day of prednisone equivalent) and dexamethasone at higher doses for more severe CNS signs and symptoms. No data support recommendations for the length of treatment; however, a 2–6-week course with close monitoring and concomitant antifungal treatment is widely accepted [7].

Corticosteroids are the only drug class that has been recognized for the treatment of IRIS so far [94]. They show an anti-inflammatory effect on most immune cells by altering the transcription of inflammatory mediators, interfering with the nuclear factor-KB, and directly enhancing the effect of the anti-inflammatory proteins [95]. An evaluation of the efficacy of corticosteroids compared to a placebo in a randomized controlled trial has only been done in TB-associated IRIS, and showed a reduced length of hospitalization and surgical procedure [96,97]. Regarding fungal infections, only case reports and small series account for the benefit of corticosteroids in fungi-associated IRIS, especially in patients with impending respiratory failure after neutrophil recovery and aspergillosis [26,98], chronic disseminated candidiasis [57], and cryptococcosis [13,58,99]. Also, despite the lack of formal diagnostic criteria for IRIS, old studies support the adjunction of corticosteroids to prevent early deterioration in patients with moderately severe *P. jiroveci* pneumonia and HIV [100]. In cryptococcal meningitis, adjunction of dexamethasone at baseline did not reduce mortality among patients with HIV, nor was it associated with a reduced incidence of IRIS in a large cohort of 451 patients [8]. On the contrary, it was correlated with more adverse effects and disability than a placebo [8]. Indeed, complications associated with the use of corticosteroids must be considered. Their non-specific immunosuppressive effect can lead to subsequent infectious complications, such as herpes virus reactivation, strongyloides hyperinfection, worsening of chronic hepatitis B, and Kaposi sarcoma progression [94,101,102]. In addition, corticosteroids can cause metabolic complications, such as dysglycemia, hypertension, and cushingoïd features, or be responsible for other adverse effects, such as worsening psychiatric disorders and drug interactions [94].

TNF-α is a pro-inflammatory cytokine required for macrophage activation and granuloma formation. The anti-TNF-α antibodies infliximab and adalimumab have been used in several case reports to reduce inflammation in IRIS. Etanercept is a soluble TNFα receptor; however, it has never been reported to be effective in IRIS treatment. Infliximab has primarily been used [103,104]; however, adalimumab has shown the ability to treat corticosteroid- and/or infliximab-resistant IRIS associated with TB and Cryptococcus [105,106,107]. Although the optimal length of treatment with such therapy has not been determined yet, the successful management of IRIS has been reported to require several months of treatment. Overall, adalimumab should probably be preferred when considering anti-TNF-α for treating IRIS, since more fungal complications have been reported with infliximab [66].

Thalidomide also acts as an immunomodulatory drug inhibiting TNFα synthesis among other cytokines (i.e. IFNγ, IL-10, and IL-12 and cyclooxygenase 2 (COX-2)) [17]. In several cases, it has been shown to be an interesting molecule for corticosteroid-dependent IRIS in cryptococcal meningitis that allows for steroid tapering [17,58,108]. The treatment duration in these studies ranged from 4 weeks to 14 months with no relapse [19].

Non-steroidal anti-inflammatory drugs (NSAIDs) inhibit cyclooxygenase, an enzyme required for prostaglandin synthesis and inflammation mediation. Many cases report the use of NSAIDs in TB-IRIS [94], and some clinical guidelines recommend their use for mild IRIS related to mycobacterial infection [109]. Nonetheless, no clinical trial supports these recommendations and data are lacking regarding fungi-associated IRIS. Moreover, nephrotoxicity is a concern with long-term use and concomitant administration of other nephrotoxic drugs, such as amphotericin B or calcineurin inhibitors.

Statins inhibit 3-hydroxy-3-methyl-glutary-CoA (HMG-CoA) reductase. It has been hypothesized that, considering the close homology between fungal and human HMG-CoA reductase, statins may have a potential antifungal effect. However, a meta-analysis of five retrospective studies showed no positive effects during fungal infections [110]. Nevertheless, statins display immunomodulatory activity promoting the Th2/Treg axis through various mechanisms [111]. Subsequently, many authors speculate that they could play a role in the management of IRIS; however, data is still lacking [111,112].

Finally, intravenous immunoglobulins have been successfully used in virus-related IRIS [113,114]; however, they have never been tried in the fungal context.

To conclude, the best-studied and most-used therapy for IRIS is corticosteroids despite their several drawbacks. Anti-TNFα, especially adalimumab, as well as thalidomide, appear to be promising in treating fungi-associated IRIS or corticosteroid-dependent IRIS. However, more studies are required. Symptomatic treatment alone, including analgesia and anti-epileptic treatment, is often sufficient to manage symptoms and should not be underestimated.

## 5. Predict and Prevent: The Cornerstone of IRIS Management Today

Since treating IRIS remains uncertain and challenging with no guidelines to rely on, patient care has been focusing on identifying risk factors and developing preventive strategies.

### 5.1. Prediction with Diagnostic Markers

The identification of risk factors mostly depends on biological markers assessed in diagnostic labs and a few clinical risk factors, especially information concerning patient treatment. Once again, cryptococcal-related paradoxical IRIS in HIV-infected patients has been the most studied situation. Therefore, our current knowledge on predictive markers is limited to these circumstances in predictive markers as well. Risk factors for cryptococcal-associated IRIS can be divided into three categories: (i) host-related factors, (ii) pathogen-related factors, and (iii) treatment-related factors. Host-related factors include various measurable immunological blood and cerebrospinal fluid (CSF) parameters. A lower pre-ART CD4 count has been demonstrated many times in bacterial- or viral-associated IRIS as well [115,116,117,118]. However, no cut-off has been established. Other blood parameters reflecting a lack of an immune response toward cryptococcosis at diagnosis appear to be relevant to predict the occurrence of IRIS. A lower plasma total IgM, a specific anti-fungal IgM (glucuronoxylomannan-IgM and β-glucan-binding IgM), and a specific IgG prior to initiation of ART were observed in patients who developed IRIS [119]. A lack of pro-inflammatory cytokines in a serum, such as TNFα, IFNγ, granulocyte-colony stimulating factor (G-CSF), and granulocyte-macrophage CSF (GM-CSF), predicted future IRIS [120]. An increase in Th2 response reflected by the IL-4 level was also associated with IRIS [120]. The use of a modified IFNγ release assay of whole blood stimulated with a cryptococcal mannoprotein has confirmed that lower IFNγ responses before ART initiation are associated with a higher risk to develop IRIS [117]. Similarly, a lack of an immune response in CSF with a decrease in the leucocytes count to ≤25 cells/µL and a reduced level of IFNγ, IL-6, IL-8, and TNF-α were associated with the development of IRIS [118,121]. In these circumstances, a global CSF protein level ≤50 mg/dL was also an independent risk factor [121]. A higher CSF ratio of CCL2/CXCL10 and CCL3/CXCL10 were also found in patients who subsequently developed IRIS [122]. CCL2 and CCL3 are chemokines known to attract monocytes, macrophages, neutrophils, and T-cells, whereas CXCL10 is only chemotactic to CXCR3+ lymphocytes (Th1 cells) [122]. Thus, an increase in the former chemokines may promote the infiltration of macrophages and neutrophils into the CSF and be responsible for IRIS.

Pathogen-related risk factors correspond to the fungal burden, which can be assessed by a serum cryptococcal antigen (CrAg) titer, the colony forming unit (CFU)/mL of CSF, and the presence of fungemia [58,118,123]. Patients with IRIS had a 4-fold higher median CrAg level pre-ART [120]. In addition, patients with a negative cryptococcal culture from a CSF sample pre-ART initiation experienced fewer CNS deterioration symptoms and a lower IRIS rate than patients with a positive culture [118].

Treatment-related risk factors include a shorter duration of antifungal treatment prior to starting ART and/or a rapid suppression of HIV viral load. Indeed, a decrease in HIV viral RNA to >2.5 log at the time of IRIS compared with RNA levels before the initiation of ART was associated with subsequent IRIS [116]. In addition, a rapid immunologic response to ART reflected by a more important rise in CD4 cells over a 6-month period was associated with IRIS [124,125]. Furthermore, some ART regimens, especially those using a boosted protease inhibitor, were a risk factor for developing IRIS [116]. Boosted protease inhibitors appear to have direct immunomodulatory effects, including anti-apoptotic effects and an increase in pro-inflammatory cytokines [116]. Recent European studies found that the use of integrase inhibitors, especially dolutegravir, increases the risk of IRIS by an odds ratio of 1.96–3.25 [126,127,128].

Regarding *Aspergillus*-related IRIS, the use of a colony-stimulating factor appears to be associated with the occurrence of IRIS in patients with invasive pulmonary aspergillosis with neutropenia [28]. Similarly, a personal case describes a severe exacerbation of CDC after G-CSF administration [129]. Concerning other fungal pathogens, no factors have been studied to predict IRIS to our knowledge. Yet, in *Aspergillus*-related IRIS, one can expect higher galactomannan titers by analogy to CrAg titers. Similarly, β-d-glucans may be higher during fungemia before chronic disseminated candidiasis. However, these statements remain hypotheses and require proof.

To conclude, no markers are yet consensual among the community, and more studies are needed to include one or several of them with proper cut-offs in standard patient care guidelines. A selection of a few of these markers, based on ease of use in the laboratory, reproducibility, price, and effectiveness to predict IRIS, should provide a strong algorithm and robust tool for stratifying patients with high, moderate, and low risk to develop IRIS.

### 5.2. Prevention by Delaying and/or Tapering Immune Restoration

IRIS depends on the critical time point when the immune system is restored. It seems that the shorter this period is, the more likely the occurrence of IRIS [25,116]. In many situations, this period cannot be controlled and only monitored to identify patients at risk to develop IRIS secondarily (i.e., neutrophil recovery, unbalanced immunosuppressive treatment in SOT). However, in HIV-infected patients, immune recovery is elicited by ART and can be adjusted. Two pioneer studies on IRIS in cryptococcal meningitis showed that initiation of ART closer to the diagnosis of the fungal disease was associated with subsequent development of IRIS [58,125]. This suggests that the inflammatory response is likely higher when the fungal burden or its remnants (i.e., antigen titers) is still substantial. Interestingly, the timing of ART appears to be more essential in *Cryptococcus*-IRIS than in TB-IRIS, in which early ART increased survival [130]. Nonetheless, this was not the case when TB meningitis was present, highlighting that CNS involvement in IRIS is the most deleterious form, and requires extra caution and specific guidelines [131].

The timing of ART initiation has been studied in four trials involving HIV-infected patients [132,133,134,135]. In the oldest one, all opportunistic infections combined, ART initiation after 2 weeks was associated with a reduced likelihood of progression or death compared to ART initiation after 6–7 weeks [132]. Opportunistic infections were mostly fungal, including 63% of *Pneumocystis* pneumonia, 12% of cryptococcal meningitis, and 4% histoplasmosis. No subanalysis was made in those groups. Surprisingly, IRIS was uncommon (7%) and was not more prevalent in the early or delayed therapy group [132]. This may be related to the smaller incidence of IRIS in *Pneumocystis* pneumonia, where corticosteroids are frequently used in severe cases. The second study focused on cryptococcal meningitis in a cohort of 54 patients in Zimbabwe, in which ART was initiated at 72-h after diagnosis versus 10 weeks later [133]. The 3-year mortality rate was significantly higher in the early ART group (88% versus 54%; p <0.006), and could be attributed to IRIS according to the authors [133]. Similarly, the third study concerned a small cohort of patients from Botswana (*n* = 27) with cryptococcal meningitis [134]. Initiation of ART within 7 days following diagnosis of fungal disease, as compared to 28 days after, was associated with a significantly increased risk of IRIS; however, there was no difference in mortality [134]. Lastly, Boulware et al. conducted an open-label randomized trial in Uganda and South Africa that enrolled HIV patients diagnosed with cryptococcal meningitis [135]. Early ART was given between 1 and 2 weeks after diagnosis, while deferred ART was given after 5 weeks. The 6-month mortality rate was significantly higher in the early ART arm (45% versus 30%; *p* = 0.03), which prematurely ended the study. The rate of IRIS was increased in the early ART arm, but not significantly different from the delayed one (20% versus 13%; *p* = 0.32). No other cause (i.e., antifungal toxicity) could explain the difference [135]. Scriven et al. attempted to explain the difference in mortality by exploring CSF macrophage activation, and found an increase of activation markers (CD206+, CD163+) on monocytes and macrophages in the early ART arm versus the delayed ART arm [136]. More data are required to determine the implications of recent ART initiation for the immune system; however, these results point to the possible involvement of innate immune response mechanisms [136].

While the IDSA guidelines had previously recommended the introduction of ART 2–10 weeks after diagnosis [7], this gap has narrowed to 4–6 weeks in newer recommendations taking into account these studies [137,138]. Though delaying ART is recommended, predictive factors should not be underestimated. Achieving a negative CSF culture prior to starting ART might be a better target to aim for to reduce IRIS risk than considering a consensual time limit, since the immune response may differ among patients. Regarding other forms of IRIS, no studies have been done concerning preventive strategies. Close monitoring of inflammation and clinical worsening is recommended to enable early care in those specific cases. 

## 6. Conclusions/Perspective

IRIS is certainly underdiagnosed and many times considered as a failure of antifungal treatment. No consensual diagnostic test is used and the diagnosis remains clinical. As far as we know, the three following criteria need to be satisfied: (1) the new appearance, or worsening, of clinical or radiographic manifestations consistent with an inflammatory process, (2) symptoms that cannot be explained by a newly acquired infection, and (3) negative culture results and/or a decrease in the fungal antigen level (BDG, galactomannan, histoplasma antigen, cryptococcal antigen…). Granuloma appear to be the histopathology hallmark, and hypercalcemia subsequent to endogenous production of 1,25 dihydroxyvitamin D by macrophages in granulomas should perhaps be sought more frequently [139]. Existing research bioassays need to be translated into clinical practice to support diagnosis. Until thorough diagnostic markers and a clear definition for fungal-associated IRIS are consensually acknowledged by the medical and scientific community, all studies included in this review ought to be considered with caution.

Regarding treatment, given the drawbacks of corticosteroid treatment, the benefit from such therapy might still be argued in cases where IRIS symptoms do not usually result in lethal complications. Furthermore, the optimal dose and length of treatment for a reasonable risk/benefit ratio need to be discussed. More studies, including randomized trials, are needed to evaluate the relevance of other anti-inflammatory drugs and to propose guidelines for the management of IRIS in fungal diseases.

The prevention of IRIS in HIV relies mostly on the timing of introduction of ART, which appears to be critical in IRIS involving the CNS compartment, such as cryptococcal IRIS resulting in significant morbidity and mortality. Other forms of IRIS, especially involving the lungs or skin, appear to be less life-threatening and have been set aside in research protocols. However, these forms of IRIS bear morbidity, a longer length of stay in the hospital, a high cost investigation, and unnecessary medications, thus requiring attention by the research community to improve the standard of care.

Our understanding of the pathogenesis of IRIS remains in its infancy. More data are available on TB-IRIS; however, additional research is needed to know if these results are applicable to fungi-associated IRIS. The heterogeneity of fungal infections and immunosuppression types contributes to the complexity of understanding IRIS occurring during fungal infections. Multifactorial approaches must be taken to understand its pathogenesis, including host genetics, fungal strain specificities, immunology, and histopathology, which could subsequently lead us to uncover new treatment options.

## Figures and Tables

**Table 1:** Clinical presentation and characteristics of fungi-associated immune reconstitution inflammatory syndrome (IRIS) by fungal species.

Pathogen	Patient Background	Symptoms	Diagnostic Test	Histopathology	Reference
*Cryptococcus* species	HIV SOT Pregnancy	Headaches, seizures, neurological deficits Lymphadenopathy, pneumonitis, chorioretinitis, skin and soft tissues lesions	**Diagnosis of exclusion** * No consensual diagnostic test Sterile CSF culture BDG- CSF PCR-	Granulomatous lesions	[7,14,15]
*Candida* species	Neutropenia (acute leukemia, lymphoma, stem cell transplant)	Fever, abdominal pain, liver and spleen enlargement MRI: mm-sized abscesses in liver, spleen, kidney, and/or lungs	**Diagnosis of exclusion** * ⍐ liver enzymes ⍐ BDG Mannan/anti-mannan antibody detection	Epithelioid granuloma, necrosis with minimal inflammatory reaction, micro-abscesses with major inflammatory reaction	[19,22,24]
*Aspergillus* species	Neutropenia (Stem cell transplant and acute leukemia)	Hypoxia, chest pain, dyspnea, hemoptysis CT scan: ⍐ pulmonary infiltrates	**Diagnosis of exclusion** * ⍗ galactomannan	Insufficiently studied	[25,26,28]
*Histoplasma capsulatum*	HIV SOT TNF-α receptor inhibitors	Hemoptysis, dyspnea, lymphadenopathy, skin nodules CT scan: pulmonary bilateral nodules and ground-glass opacities	**Diagnosis of exclusion** * ⍗ *Histoplasma* serum antigen Sterile culture	Well-formed granulomatous inflammation	[29,30,31,32,33]
*Pneumocystis jiroveci*	HIV Corticosteroid-treated patients	Fever, cough, dyspnea, night sweat	**Diagnosis of exclusion** *	Organizing pneumonia: organizing granulation tissue	[39,40,41,42,44]

* Exclusion of other diagnosis: microbial progression, other opportunistic infection, tumors, a drug-related adverse effect. SOT, Solid Organ Transplant; BDG, (1-3)-β-D-glucan; CSF, cerebral spinal fluid; HIV, human immunodeficiency virus.
